# Intranasal Peptide-Based FpvA-KLH Conjugate Vaccine Protects Mice From *Pseudomonas aeruginosa* Acute Murine Pneumonia

**DOI:** 10.3389/fimmu.2019.02497

**Published:** 2019-10-23

**Authors:** Emel Sen-Kilic, Catherine B. Blackwood, Dylan T. Boehm, William T. Witt, Aaron C. Malkowski, Justin R. Bevere, Ting Y. Wong, Jesse M. Hall, Shelby D. Bradford, Melinda E. Varney, Fredrick Heath Damron, Mariette Barbier

**Affiliations:** ^1^Department of Microbiology, Immunology, and Cell Biology, West Virginia University School of Medicine, Morgantown, WV, United States; ^2^Vaccine Development Center at West Virginia University Health Sciences Center, Morgantown, WV, United States

**Keywords:** FpvA, peptide-based vaccines, *Pseudomonas aeruginosa*, infection and immunity, vaccine, cystic fibrosis, mucosal immunity, iron acquisition

## Abstract

*Pseudomonas aeruginosa* is an opportunistic pathogen causing acute and chronic respiratory infections associated with morbidity and mortality, especially in patients with cystic fibrosis. Vaccination against *P. aeruginosa* before colonization may be a solution against these infections and improve the quality of life of at-risk patients. To develop a vaccine against *P. aeruginosa*, we formulated a novel peptide-based *P. aeruginosa* subunit vaccine based on the extracellular regions of one of its major siderophore receptors, FpvA. We evaluated the effectiveness and immunogenicity of the FpvA peptides conjugated to keyhole limpet hemocyanin (KLH) with the adjuvant curdlan in a murine vaccination and challenge model. Immunization with the FpvA-KLH vaccine decreased the bacterial burden and lung edema after *P. aeruginosa* challenge. Vaccination with FpvA-KLH lead to antigen-specific IgG and IgM antibodies in sera, and IgA antibodies in lung supernatant. FpvA-KLH immunized mice had an increase in recruitment of CD11b^+^ dendritic cells as well as resident memory CD4^+^ T cells in the lungs compared to non-vaccinated challenged mice. Splenocytes isolated from vaccinated animals showed that the FpvA-KLH vaccine with the adjuvant curdlan induces antigen-specific IL-17 production and leads to a Th17 type of immune response. These results indicate that the intranasal FpvA-KLH conjugate vaccine can elicit both mucosal and systemic immune responses. These observations suggest that the intranasal peptide-based FpvA-KLH conjugate vaccine with curdlan is a potential vaccine candidate against *P. aeruginosa* pneumonia.

## Introduction

*Pseudomonas aeruginosa* is one of the leading opportunistic Gram-negative pathogens responsible for life-threatening respiratory infections (1). High adaptability and increasing prevalence of multidrug-resistant *P. aeruginosa* poses a significant threat for at-risk patients (2). People with compromised immunity, chronic obstructive pulmonary disease, cystic fibrosis (CF), or those who receive immunosuppressive therapies are particularly susceptible to *P. aeruginosa* infections (1). *P. aeruginosa-*associated chronic lung infections are one of the main contributors to loss of pulmonary function, which is the primary cause of death in patients with CF (3, 4). Based on the Cystic Fibrosis Foundation 2017 annual report, the median age at first detection of *P. aeruginosa* infection is 5.2 years in CF patients (3). Although eradication is possible early in life, once the airway of a CF patient becomes chronically colonized, it is difficult to treat *P. aeruginosa* infections (5). Therefore, therapies that prevent or delay the colonization of *P. aeruginosa* in CF airways have the potential to increase pulmonary function, and thus improve the longevity and quality of a CF patient's life. An effective vaccine against *P. aeruginosa* could provide a solution against infections caused by this bacterium in CF patients, as well as in other at-risk populations.

Recent efforts in *P. aeruginosa* vaccine development have focused on subunit vaccines based on virulence mechanisms of *P. aeruginosa*, such as lipopolysaccharide (LPS), flagella, alginate, and outer-membrane proteins (OMPs) (6). While a number of these vaccines showed promising results in clinical studies, no vaccine against *P. aeruginosa* has been approved for human use (7). Lack of efficacy has been attributed to serotype variation of LPS and flagella, as well as to the difficulty of performing clinical trials in *P. aeruginosa* at-risk populations (6). Although there is no vaccine available for clinical use, research efforts focused on the use of OMPs as vaccine antigens are highly promising. OMPs are surface-exposed, often more conserved across strains with varying LPS serotypes, and can be recognized by the immune system during natural infection, which makes them potential vaccine candidates (8, 9). In addition, we propose that these proteins need to be expressed during infection to be relevant as antigens. In previous studies performed by our laboratory, we identified *P. aeruginosa* genes expressed during acute murine pneumonia (10). From this study, we observed that genes associated with iron acquisition were significantly up-regulated in *P. aeruginosa* during *in vivo* acute lung infection and decided to further examine their potential use as vaccine antigens.

Iron is an essential nutrient for *P. aeruginosa* virulence and survival in the host (11). During infection, *P. aeruginosa* competes for iron with the host using its siderophores and two other systems for heme uptake (12). Iron availability is correlated with *P. aeruginosa* persistence in the lungs of CF patients (13), and iron acquisition systems are expressed within CF sputum (14). Each iron acquisition system of *P. aeruginosa* includes an outer-membrane receptor located on the surface of the organism. Therefore, we hypothesize that these receptors involved in iron acquisition in *P. aeruginosa* can be used as antigens to generate a subunit *P. aeruginosa* vaccine. Among these receptors, the ferripyoverdine receptor FpvA is involved in siderophore-mediated iron uptake (15). In addition, our laboratory observed that the gene encoding this receptor is highly expressed *in vivo* during acute murine pneumonia (10). *P. aeruginosa* uses FpvA to bind the high-affinity siderophore pyoverdine to capture iron from the host environment and translocate it to the cell cytoplasm through a TonB-dependent system (15). Studies from Wu et al. and Liu et al. have previously identified and tested FpvA as a potential antigen against *P. aeruginosa* (16, 17). FpvA displays numerous characteristics often associated with protective antigens: (1) FpvA is present on the surface of the bacterium (18), (2) is expressed during infection (10, 17, 19), (3) is important for bacterial homeostasis and virulence (12, 17, 20, 21), and (4) is present in the majority of *P. aeruginosa* strains including isolates from CF patients (22, 23). In addition, iron acquisition receptor-based vaccines are protective against *E. coli, Klebsiella* spp., *Salmonella enterica serovar* Enteritidis, *Neisseria gonorrhoeae, Neisseria meningitidis, Staphylococcus aureus*, and *Haemophilus parasuis* in different animal models (24–31). Therefore, the iron acquisition receptor FpvA has the potential to be used as an antigen against *P. aeruginosa*. However, purification of FpvA and maintenance of the proper folding of the protein during the purification process is challenging since FpvA is an OMP (17, 32). To circumvent this problem, we selected smaller peptides based on the outer-membrane regions of this protein that are more soluble and may be used as a substitute for the whole protein. We hypothesize that a peptide-based vaccine containing outer-membrane regions of FpvA can provide protection against *P. aeruginosa* respiratory infections.

In this study, we generated a vaccine containing a cocktail of peptide antigens based on the outer-membrane regions of FpvA. These peptides were covalently attached to the carrier molecule keyhole limpet hemocyanin (KLH) to increase immunogenicity. Previous studies showed that both Th1 and Th17 associated immune responses are important to reduce the severity of *P. aeruginosa* lung infections (33, 34). Therefore, the vaccine was formulated with the β-glucan adjuvant curdlan, a known inducer of Th1/Th17 immune responses (35). In addition, numerous pathogens, including *P. aeruginosa*, use mucosal surfaces as the initial site of entry into the human body (36, 37). Therefore, having both systemic and mucosal immune responses against *P. aeruginosa* is often thought to be crucial for protection. Intranasal vaccination induces both systemic and mucosal immune responses in the respiratory tract (38). Thus, the vaccine was administered intranasally. By performing vaccination and challenge studies in a murine model of acute pneumonia, we evaluated the vaccine's immunogenicity and protective potential. We determined that intranasal peptide-based FpvA-KLH conjugate vaccination is effective to decrease the bacterial burden in the airways and lung edema following intranasal challenge. We showed that intranasal vaccination with FpvA-KLH peptides and curdlan as an adjuvant elicits both systemic and mucosal humoral and cellular immune responses. Overall, this study supports that in a murine model, the intranasal administration of a peptide-based FpvA conjugate vaccine is an efficient vaccine strategy against acute pneumonia caused by *P. aeruginosa* and could be pursued as a potential human vaccine candidate.

## Methods

### Bioinformatic Design and Selection of FpvA Peptide Antigens

Peptides were designed using a multi-step approach: (1) The crystal structure of the FpvA protein was obtained from the RCSB Protein Data Bank (PDB) ID:2IAH (39). The extracellular regions of FpvA were determined from deposited transmembrane regions in the Orientations of Proteins in Membranes (OPM) database (40). (2) The protein sequence of FpvA was analyzed to identify hydrophilic regions based on the Kyte-Doolittle scale using CLC Genomics Workbench 7.5.1 (41) and to predict linear B cell epitopes using the BepiPred algorithm (42). Based on the hydrophilicity and B cell linear epitope prediction, candidate peptides overlapping with FpvA extracellular regions and with a length comprised between 20 and 30 amino acids were selected. (3) The candidate peptides were screened for homology to other proteins using NCBI-BLASTP (protein-protein BLAST) (43). Peptides with a percentage of identity higher than 50%, when compared to non-pathogenic commensal bacteria or human protein sequences, were excluded from the study to avoid any undesired cross-species reactivity. (4) Four peptides were selected and synthesized by Biomatik USA with >85% w/w purity. A cysteine residue was added on the N-terminus of the peptide sequence to allow for conjugation to a carrier protein. (5) Each peptide was conjugated to Keyhole Limpet Hemocyanin (KLH). The peptides and conjugates were validated using mass spectrometry and High performance liquid chromatography (HPLC) by the manufacturer. Chimera 1.10.2 (44) was used to visualize selected peptides and FpvA protein.

### Bacterial Strains

The following strains were used: *P. aeruginosa* PAO1 “Vasil” (Dr. Michael L. Vasil, University of Colorado), PAO1 “Washington” (Dr. Colin Manoil, University of Washington), PAO1 “Spain” (Dr. Sebastian Alberti, University of the Balearic Islands), PA14 (Dr. Frederick M. Ausubel, Harvard University), and CF clinical isolates (45) (Dr. Robert Ernst, University of Maryland). *P. aeruginosa* PAO1 “Vasil” strain was used for vaccination and challenge. *P. aeruginosa* was grown for 16 h at 37°C on Pseudomonas Isolation Agar (PIA) (Becton Dickinson) unless otherwise specified.

### Vaccine Formulation

FpvA and FpvA-KLH peptides were resuspended in 125 mM NaOH (Fisher Scientific). The unconjugated FpvA peptide vaccine was prepared using a mix of 35 μg of each FpvA peptide in PBS. The FpvA-KLH conjugate vaccine dose consisted of a mix of 35 μg of each FpvA-KLH peptide in PBS. The whole-cell vaccine (WCV) was prepared using *P. aeruginosa* PAO1 “Vasil.” The strain was grown as described above. Swabbed bacteria were resuspended in Phosphate Buffered Saline (PBS), and heat-killed at 60°C for 1 h. The efficiency of heat-killing was verified by plating on PIA. Each WCV dose consisted of 4–5 × 10^7^ heat-killed colony-forming unit (CFU) of *P. aeruginosa* in PBS. All vaccines were prepared in a final volume of 20 μl and contained 100 μg of curdlan (Sigma Aldrich) as an adjuvant unless otherwise specified. The curdlan-only control consisted of 100 μg of curdlan in PBS.

### Murine Immunization

Groups of 6-week-old outbred female CD-1 mice (Strain code:022, Charles River) were anesthetized by intraperitoneal (IP) injection of a total of 0.2 ml of ketamine (7.7 mg/kg) (Patterson Veterinary) and xylazine (0.77 mg/kg) (Patterson Veterinary) in 0.9% saline according to the Institutional Animal Care and Use Committee guidelines. Mice were immunized intranasally with adjuvant only, unconjugated FpvA peptides, FpvA-KLH conjugates, or WCV at day 0 followed by a booster at day 21. The experiments were performed in accordance with the National Institutes of Health Guide for the care and use of laboratory animals. The protocols used were approved by West Virginia University Institutional Animal Care and Use Committees (WVU-ACUC protocol 1606003173).

### Blood Sample Collection

Blood samples were collected from the tail vein or submandibular vein of each mouse at days 0, 7, 14, and 20 post-vaccination. The samples were left at ambient temperature for 30 min to allow the blood to coagulate and centrifuged for 2 min at 17,900 *x g*. Serum was collected and stored at −80°C.

### ELISA

The antibody titers were assayed by ELISA in Costar^®^ 96 well high-binding microtiter plates (Corning Incorporated). The microtiter plates were coated with a volume of 50 μl/well of 2 × 10^7^ CFU of heat-killed *P. aeruginosa* PAO1, 500 ng of individual FpvA peptides, or a mix of all peptides (125 ng of each peptide; 500 ng total) overnight at 4°C. After coating, the plates were washed three times with PBS with 0.05% Tween 20 (Fisher Scientific) (PBS-T) and blocked with 2% Bovine Serum Albumin (BSA) (Bioworld) in PBS overnight at 4°C. Blocked plates were washed three times with PBS-T. The samples were prepared in 2% BSA in PBS. The serum samples were serially diluted from 1:50 to 1:25,600, and the lung supernatants were serially diluted from 1:1 to 1:256. After 1 h of incubation at 37°C, the plates were washed four times with PBS-T and incubated with anti-IgG, -IgM, -IgG1, -IgG2a or -IgG2b, -IgA alkaline-phosphatase conjugated goat anti-mouse antibodies (SouthernBiotech) at 1:2,000 per well for 1 h at 37°C. Plates were then washed five times with PBS-T and incubated with Pierce *p*-Nitrophenyl Phosphate (PNPP) (Thermo Fisher Scientific) for 30 min following the manufacturer's instructions. The absorbance of the plates was read at 405 nm using SpectraMax i3 (Molecular Devices LLC). Antibody titers were determined as the highest dilution of the serum with a signal two times above the average of blanks. To measure the serum IgG response, eleven CF clinical isolates described by Burns et al. (45), different *P. aeruginosa* PAO1 strains, and the PA14 strain (46) were grown at 37°C on minimal growth medium M9 (Teknova) overnight. The following day, the isolates were diluted 1:20 and grown exponentially on M9 media until they reached OD_600_ = 0.25. The Costar^®^ 96 well high-binding microtiter plates (Corning Incorporated) were coated with 50 μl/well of 2 × 10^7^ CFU of these cultures. Plates were then incubated with pooled sera from NVC and FpvA-KLH vaccinated challenged mice (1:50 diluted in PBS). Anti-*P. aeruginosa* antibodies present in these samples were then detected using the procedure was done as described above. To characterize the lung IgA response, PAO1 and CF clinical isolates were grown and Corning^TM^ 96-well white high-binding microtiter plates (Corning Incorporated) were coated as described above. Plates were then incubated with pooled lung supernatants from NVC and FpvA-KLH vaccinated challenged mice (1:16 diluted in PBS). For detection, an anti-IgA horseradish peroxidase conjugated goat anti-mouse secondary antibody (SouthernBiotech) (1:4,000 per well) and the SuperSignal™ ELISA Femto Substrate (Thermo Fisher Scientific) were used. The chemiluminescence signal of the plates was detected using Synergy HTX Multi-Mode Reader (BioTek).

### Bacterial Challenge and Respiratory Tissue Processing at 16 h Post-challenge

The bacterial challenge was performed at day 34, and mice were dissected at 16 h post-challenge. Anesthetized mice were challenged intranasally with 20 μl of the PAO1 strain (6 × 10^7^ CFU in PBS unless otherwise specified) grown as described above. As a vehicle control, 20 μl of PBS was administered to the adjuvant-only group (non-vaccinated non-challenged, NVNC). Mice were euthanized by IP injection of 390 mg pentobarbital/kg (Patterson Veterinary) in 0.9% NaCl. The blood was collected by cardiac puncture after euthanasia. The lungs of each mouse were aseptically removed and weighed. Nares were flushed with 1 ml of sterile PBS to collect the nasal washes (NW). Lungs were homogenized in 1 ml of PBS using a glass Dounce homogenizer. One hundred microliters of homogenized lung samples and NW from each mouse were then serially diluted and plated on PIA plates to determine viable CFU counts. To measure the cytokine response, 200 μl of lung homogenate from each mouse were pelleted by centrifugation. The lung supernatant was collected and stored at −80°C until cytokine analysis. The remaining lung homogenates were further analyzed by flow cytometry.

### Purification of FpvA Protein

The gene *fpvA* was amplified by PCR from PAO1 “Vasil” genomic DNA. Primers were designed to include a SacI restriction site on the forward primer, and a His6 tag and a HindIII restriction site on the reverse primer to facilitate cloning and purification (Table S1). The PCR fragment was first cloned into pCR4-TOPO (Invitrogen) to generate pCR4-TOPO-*fpvA*, and resulting clones were confirmed using Sanger sequencing. pCR4-TOPO-*fpvA* was then digested with SacI and HindIII and ligated into pHERD20T (47) digested with the same enzymes. Resulting plasmids were transformed into *Escherichia coli* strain Clear Coli (Lucigen), and correct insertion of *fpvA* in pHERD20T was confirmed by Sanger sequencing. For protein purification, pHERD20T and pHERD20T*fpvA* were grown overnight in Lysogeny Broth (LB, Miller formulation) supplemented with 100 μg/ml carbenicillin (Fisher Scientific) at 37°C. Cultures were then diluted 1:100 in fresh LB and grown for 6 h at 37°C. Protein expression was then induced by addition of 0.1% w/v L-arabinose (Sigma-Aldrich) for 18 to 24 h at 37°C. Cells were harvested by centrifugation at 5,000 *x g* for 10 min at 4°C. Cell pellets were washed with PBS and centrifuged at 5,000 *x g* 10 min 4°C. Cells were lysed for 30 min on ice with PBS containing 1 mM MgCl_2_, protease inhibitors (Halt protease cocktail EDTA free) (Thermo Fisher Scientific) and 160 μg/ml lysozyme (Sigma-Aldrich). Lysates were then sonicated twice for 30 s on ice and centrifuged at 20,000 *x g* for 15 min at 4°C. Pellets were solubilized with 1.5% w/v dodecyl-beta-D-maltoside (MP Biochemicals) in PBS. Samples were centrifuged 20,000 *x g* for 15 min at 4°C and supernatants transferred to HisPur Cobalt columns (Thermo Fisher Scientific). Elutions were pooled and dialyzed with 10 K molecular weight cut-off SnakeSkin Dialysis Tubing (Thermo Fisher Scientific) in water overnight at 4°C. Samples were then concentrated using an Amicon 50 kDa Nominal Molecular Weight Limit (Millipore). Protein concentrations were measured using bicinchoninic acid assay (BCA) total protein kit (Thermo Fisher Scientific).

### Western Blot Detection of FpvA-Specific Antibodies

Five μg of purified FpvA protein were resuspended in Laemmli buffer (Bio-Rad), boiled for 5 min, loaded into each well, and resolved by sodium dodecyl sulfate-polyacrylamide gel electrophoresis (SDS-PAGE). Separated proteins were transferred to a previously rehydrated PVDF membrane (Bio-Rad). After the transfer, the membrane was blocked with 5% w/v skim milk (BD Diagnostic Systems) in PBS-T at 4°C overnight. The following day, the membrane was incubated with pooled murine sera from the FpvA-KLH (*n* = 5) and NVC (*n* = 5) groups at a dilution of 1:2,000, or with anti-His antibodies (Proteintech Group) at 1:5,000 in 5% w/v skim milk in PBS-T for 16 h. The membrane was washed three times with PBS-T and incubated with anti-mouse IgG antibodies conjugated with HRP (Immunoreagents) at 1:5,000 in 5% w/v skim milk in PBS-T for 1 h. The membrane was rewashed three times with PBS-T and finally developed using SuperSignal West Femto Maximum Sensitivity Substrate (Thermo Fisher Scientific). Chemiluminescence signal was detected using a Chemidoc Touch Imaging System (Bio-Rad).

### Opsonophagocytic Killing Assay

*P. aeruginosa* PAO1 was grown in 5 ml of LB overnight. The following day, the suspension was diluted at 1:100 dilution in fresh LB and exponentially grown to OD_600_ = 0.3. The cells were pelleted and re-suspended in 5 ml of minimal essential medium (MEM) (Corning Incorporated) with 1% BSA (MEM-BSA) (Bioworld). The suspension was diluted in MEM-BSA to obtain a final concentration. J774A.1 macrophages (ATCC) were grown in DMEM medium (Corning Incorporated), harvested and resuspended in MEM-BSA. Mice sera from NVNC, WCV, and FpvA-KLH groups were used for opsonophagocytic assays at a concentration of 2x the titer for anti-*P. aeruginosa* antibodies for each serum. Sera of each group were incubated for 30 min at 56°C to inactivate complement activity. *P. aeruginosa* PAO1 (2.5 × 10^5^ CFU/ml), macrophages (2.5 × 10^5^ cells/ml), and serum sample were incubated together in microcentrifuge tubes by tumbling end-over-end on a rotator at 37°C for 1.5 h. As control, the same amount of NVNC control serum was used for each WCV and FpvA-KLH serum sample. After the incubation period, samples were serially diluted and plated on PIA. The percentage of bacterial killing was calculated by comparing the number of bacteria killed in samples incubated with WCV or FpvA-KLH sera to the NVNC control serum. The percent killing was calculated relative to NVNC control serum. Percent Kill = [{(CFU counts from the same amount of NVNC serum) – (CFU counts from vaccinated challenged serum)}/(CFU counts from the same amount of NVNC serum) × 100]. Serum from each mouse was evaluated using technical duplicates.

### Hematologic Analysis

To measure the number of white blood cells in blood upon challenge, 200 μl of blood were collected into BD Microtainer^®^ Blood Collection Tubes (BD Biosciences). Blood samples were analyzed immediately using an Hemavet 950 FS (Drew Scientific). The results were compared to a mouse blood standard (Drew Scientific).

### Flow Cytometry Sample Preparation and Analysis

Flow cytometry was performed on lung and spleen samples. Lung samples were prepared as described above. Lung homogenates were diluted with 4 ml of PBS and the suspension was strained using a 100 μm pore cell strainer (VWR International). The spleens from mice challenged with 10^7^ CFU were collected at day 7 post-challenge, diluted with 4 ml RPMI 1640 medium (Thermo Fisher Scientific) with 10% v/v fetal bovine serum (FBS) and 0.5% w/v penicillin-streptomycin (Corning) (RPMI complete medium), and homogenized gently through a disposable 100 μm pore cell strainer. Samples were centrifuged at 1,000 *x g* for 5 min, and supernatants were removed. Red blood cells were lysed using Pharm Lyse (BD Biosciences) for 2 min at 37°C. The remaining cells were centrifuged at 1,000 *x g* for 5 min. The pellets were resuspended in PBS with 1% v/v FBS for Fc receptor blocking and incubated on ice for 15 min. Lung cells were stained with myeloid and resident memory cell surface markers (Table S2). Each cell suspension sample was incubated with the antibody cocktails for 1 h at 4°C in the dark. Cell suspension samples were pelleted and resuspended in PBS. Spleen cells were stained with specific T cell surface markers and intracellular transcription factor markers using Pharmingen™ Transcription Factor Buffer (BD Biosciences) (Table S2). The cell surface staining was performed as described above. The cells were then pelleted, fixed, and permeabilized with 1 ml of Fix/Perm buffer (BD Biosciences) for 50 min at 4°C. The fixed and permeabilized cells were washed with 1 ml of Perm/Wash buffer (BD Biosciences) twice before intracellular staining for 50 min at 4°C in the dark. Cell suspension samples were pelleted, washed with Perm/Wash buffer and resuspended in PBS. Samples were processed using a LSR Fortessa flow cytometer (BD Biosciences) and analyzed using FlowJo v10 (FlowJo, LLC). The flow cytometry gating strategy for myeloid cells was adapted from Misharin et al. (48) (Figure S1).

### Cytokine Analysis

To measure the cytokine response, the concentration of IFN-γ, IL-1β, IL-2, IL-4, IL-5, IL-6, KC/GRO, IL-10, IL-12p70, TNF-α, and IL-17 in the lung were determined by quantitative sandwich immunoassays using the Meso Scale Diagnostics V-PLEX Plus Proinflammatory Panel 1 Mouse Kit (K15048G-1) and Mouse IL-17 Ultra-Sensitive kits (K152ATC-1), following the manufacturer's instructions. The electrochemiluminescence signal was detected using the MSD Multi-Array Imaging Platform (Meso Scale Diagnostics). Lung supernatants from NVNC, NVC, and FpvA-KLH groups were diluted 1:5 and 1:300 to avoid saturation of the signal.

### ELISpot

ELISpot assays were performed to determine the number of antigen-specific splenocytes in vaccinated animals secreting IL-17 and IFN-γ in response to antigen stimulation. For these experiments, a FpvA-KLH vaccine was also formulated with a mixture of 35 μg of each FpvA-KLH peptide and 62.5 μg of alhydrogel (Invivogen) in PBS. EMD Millipore Multiscreen 96-well assay (Thermo Fisher Scientific) plates were pre-wetted with 15 μl of 35% v/v ethanol for 1 min. The plates were washed five times with 200 μl of sterile water and coated with anti-IL-17 (5 μg/ml) (Mabtech) and anti-IFN-γ (5 μg/ml) (BD Biosciences) antibodies overnight at 4°C. Splenocytes were isolated from immunized and boosted CD-1 mice as described above. The spleen of each mouse was collected at day 34 and homogenized using a 100 μm sterile cell strainer (VWR International). The splenocytes were counted using trypan blue (Life Technologies) with a Countess II FL Automated Cell Counter (Invitrogen). The cells were diluted to 10^6^ cells/ml using RPMI complete medium. The splenocytes were then aliquoted in duplicate and incubated with RPMI complete medium only, or RPMI complete medium containing 10^7^ heat-killed *P. aeruginosa* using pre-wetted, antibody-coated plates. The splenocytes from non-vaccinated control mice were also incubated with RPMI complete medium containing 50 ng/ml Phorbol 12-myristate 13-acetate (PMA) (Fisher Scientific) as a positive stimulant control. IL-17 antibody-coated plates were incubated for 72 h and IFN-γ antibody-coated plates were incubated for 16 h at 37°C with 5% CO_2_. After the incubation, the wells were washed and incubated with biotinylated anti-IL-17 (0.25 μg/ml), or anti-IFN-γ (2 μg/ml) secondary antibodies for 2 h at room temperature according to the manufacturer's instructions. The wells were then rewashed, incubated with streptavidin-alkaline phosphatase (Mabtech) for 1 h at room temperature, and developed using a filtered substrate solution of BCIP/NBT (Mabtech). ELISpot plates were imaged using an Olympus MVX10 Microscope. The images were processed with FFT Bandpass filter, converted to binary images and counted automatically for particles bigger than 10 pixel^2^ using Image J software v.1.52a (49).

### Histological Analysis

For these experiments, mice were vaccinated as described above and challenged with 2 × 10^8^ CFU of *P. aeruginosa* PAO1. The lungs were collected from vaccinated mice at 16 h post-challenge, perfused, and fixed with 4% v/v formalin (Fisher Scientific). The samples were sectioned and stained with hematoxylin and eosin (H&E) by the WVU Pathology Department. A total of 10 images per 2 slides in each group were imaged at 10x and 40x magnification on an EVOS XL Cell Imaging System (Thermo Fisher Scientific).

### Statistical Analysis

All statistical analyses were performed using the software Prism version 7 (GraphPad). Comparisons between two groups were performed using an unpaired Student's *t*-test. Comparisons between three or more groups were analyzed by one-way analysis of variance (ANOVA) followed by a Tukey's multiple comparison test for data that follows a normal distribution. Statistical analysis for non-parametrical data was performed using the Kruskal-Wallis test with Dunn's multiple comparison test. For statistical analysis of non-parametric samples for which no signal was detected (i.e., NVNC and NVC serum titers), comparisons were made to the hypothetical value of 1 using a Wilcoxon signed-ranked test to compare the antibody titer responses. One sample *t*-test was used for comparison of opsonophagocytic killing assays.

## Results

### FpvA-KLH Peptide Vaccination Leads to FpvA-Specific Antibody Production in a Murine Model of Vaccination

**Figure 1:**
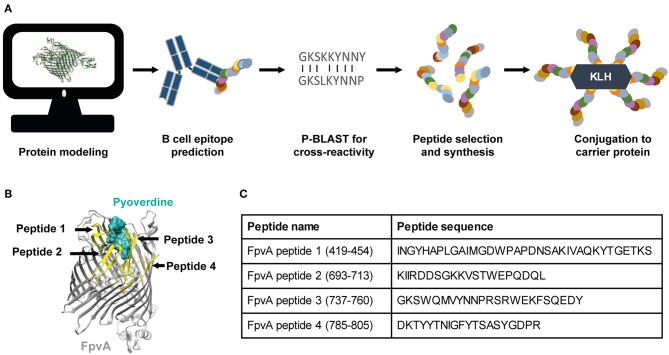
Vaccine design and selection of the FpvA peptides. **(A)** Schematic representation of the FpvA peptide design process. **(B)** Structure of FpvA protein (gray) bound to pyoverdine (ocean green) (PDB ID: 2IAH). The peptides used in this study are highlighted in yellow. **(C)** Peptide sequences and amino acid residue numbers of the selected FpvA peptides.

To develop a vaccine against *P. aeruginosa* using the OMP FpvA, we designed and selected peptides based on the outer-membrane regions of the protein. The workflow of peptide design is shown in Figure 1A. The selected peptides were synthesized and conjugated to the carrier protein, keyhole limpet hemocyanin (KLH), to increase their immunogenicity. Peptide position and sequence information are shown in Figures 1B,C.

**Figure 2:**
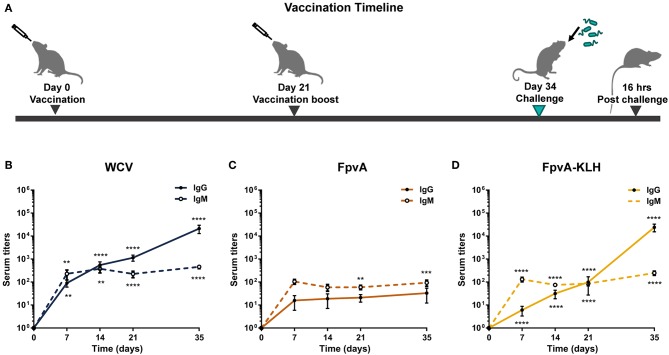
Experimental design and antibody responses over time. **(A)** Schematic diagram of the vaccination timeline and overall experimental design. Mice were vaccinated with FpvA peptides, FpvA-KLH conjugates, WCV or adjuvant only (NVNC) and received a booster of the same vaccine at day 21. Mice were challenged with *P. aeruginosa* PAO1 by intranasal instillation at day 34. IgG and IgM anti-PAO1 antibodies in WCV vaccinated mice (*n* = 14) **(B)**, anti-unconjugated FpvA peptide antibodies in FpvA-vaccinated mice (*n* = 13) **(C)**, or in FpvA-KLH vaccinated mice (*n* = 14) **(D)** collected at day 7, day 14, day 20, and day 35. No antibody response was detected in NVNC animals, the comparisons were made to the hypothetical value of 1 using Wilcoxon signed-ranked test. The asterisks refer to statistical significance: ^**^p ≤ 0.01, ^***^p ≤ 0.001, ^****^p ≤ 0.001. Error bars are mean ± SEM values.

**Figure 3:**
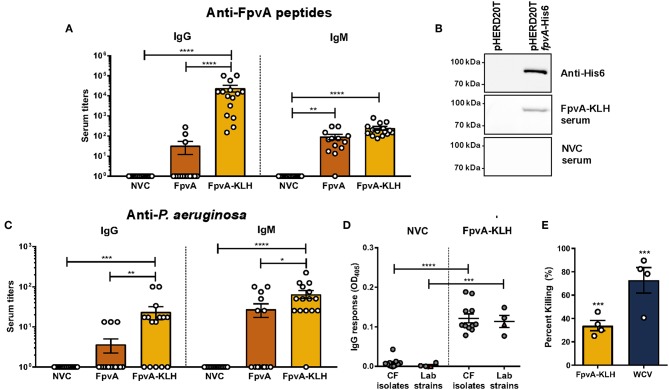
Antibodies elicited by FpvA-KLH peptide vaccination can recognize the FpvA peptides, FpvA protein, and *P. aeruginosa*. **(A)** IgG and IgM antibody titers of NVC, FpvA peptides only, and FpvA-KLH vaccinated and challenged groups. Unconjugated FpvA peptides were used as antigen. **(B)** Western blot analysis of pooled NVC and FpvA-KLH (*n* = 5) sera against purified FpvA protein expressed in *E. coli* using the vector pHERD20T*fpvA-*His6. pHERD20T is used as a vehicle control. **(C)** IgG and IgM antibody titers of NVC, FpvA peptides only and FpvA-KLH vaccinated and challenged groups. Heat-killed whole-cell *P. aeruginosa* PAO1 was used as antigen. In **(A,C)**, each circle represents data from one mouse of NVC (*n* = 14), WCV (*n* = 14), FpvA (*n* = 13), and FpvA-KLH (*n* = 14) vaccine groups. Data points represent the mean of three independent experiments. The *p*-values were calculated using a Kruskal Wallis test with Dunn's multiple comparison test. **(D)** IgG response of pooled sera from NVC and FpvA-KLH vaccinated challenged mice using *P. aeruginosa* cystic fibrosis isolates (*n* = 11) and lab strains (PAO1 and PA14) (*n* = 4) as antigens. Each circle represents different *P. aeruginosa* strains, triangles show the PAO1 strain used for infection. The *p*-values were calculated using unpaired Student's *t*-test. All data represent titers collected at 16 h post-challenge. **(E)** Percentage of opsonophagocytic killing activity of FpvA-KLH and WCV sera against *P. aeruginosa* PAO1. Percentage was calculated relative to NVNC control. The *p*-values were calculated using a one-sample *t*-test. The asterisks refer to statistical significance: ^*^p ≤ 0.05, ^**^p ≤ 0.01, ^***^p ≤ 0.001, ^****^p ≤ 0.0001. Error bars are mean ± SEM values.

To determine the immunogenicity of the peptides, we formulated them as vaccines with the adjuvant curdlan (16). We used *P. aeruginosa* heat-inactivated whole cell vaccine (WCV) with curdlan as a positive control. Curdlan alone (non-vaccinated challenged, NVC) was used as vehicle control. Cocktails of unconjugated FpvA peptides, FpvA-KLH peptide conjugates, and control groups were intranasally administered to 6-week-old female CD-1 mice (Figure 2A). To determine the immunogenicity of each vaccine, IgG and IgM levels were measured over time. Using this experimental design, we observed as expected that our positive control WCV led to an increase in the production of anti-*P. aeruginosa* IgM, followed by a class-switch to IgG over time (Figure 2B). The unconjugated FpvA peptides elicited low anti-FpvA peptide-specific IgM antibody titers that did not result in significant isotype switching to IgG (Figure 2C). This suggests that the unconjugated FpvA peptides are poorly immunogenic and fail to induce a significant IgG antibody response over time. In the FpvA-KLH vaccinated mice, we observed a significant production of anti-FpvA peptide-specific IgM antibodies. In addition, we observed isotype switching to IgG in the FpvA-KLH group (Figure 2D). We also observed that mice immunized with the FpvA-KLH vaccine resulted in significant increase in anti-FpvA peptide-specific IgG compared to unconjugated FpvA peptides at day 35 post-vaccination (1,000-fold) (Figure 3A). Compared to the NVC group, immunization with FpvA-KLH lead to the significant production of anti-FpvA peptide-specific IgG and IgM (Figure 3A). While the mice were immunized with a mix of four FpvA peptides conjugated to KLH, anti-peptide 2 antibodies accounted for most of the IgG serum titers, and anti-peptide 4 antibodies were detected in three out of nine mice (Figure S2).

We observed that vaccination with FpvA-KLH could induce an antibody response that recognizes the FpvA peptides. However, this response is unlikely to be protective if these antibodies do not recognize the FpvA protein or the whole bacterium. To determine if antibodies generated by vaccination with FpvA-KLH could recognize the full-length protein, we performed Western blot analysis with the purified recombinant FpvA protein. The results indicate that the serum from FpvA-KLH vaccinated mice, but not NVC mice, recognizes the purified recombinant FpvA protein (Figure 3B). Finally, we tested whether the antibodies elicited by FpvA-KLH vaccination could recognize the whole bacteria. We detected a significant amount of IgG and IgM against *P. aeruginosa* PAO1 in the FpvA-KLH vaccinated mice compared to NVC (Figure 3C). Similar to the data obtained in Figure 3A, conjugation of the peptides with KLH resulted in significant increase in IgG (100-fold) and in IgM antibody response (10-fold) compared to the unconjugated FpvA peptides (Figure 3C). Immunization with the unconjugated FpvA peptides did not produce a significant antibody response against *P. aeruginosa* PAO1 compared to NVC (Figure 3C).

To characterize whether sera from FpvA-KLH vaccinated mice could recognize other *P. aeruginosa* strains, we performed ELISA experiments with 11 *P. aeruginosa* clinical isolates from the sputum and bronchoalveolar lavage of CF patients (45). We compared the serum IgG response to various *P. aeruginosa* laboratory-adapted strains, including different *P. aeruginosa* PAO1 and PA14 strains (46). The data obtained indicate that the serum from FpvA-KLH peptide-vaccinated mice can bind to CF isolates as well as the lab strains above the background threshold, suggesting that these antibodies can also recognize clinical *P. aeruginosa* isolates (Figure 3D). These data indicate that vaccination with FpvA-KLH peptides triggers the production of antibodies that recognize the unconjugated FpvA peptides, purified recombinant FpvA protein, as well as different *P. aeruginosa* strains.

Finally, to determine the role of these antibodies in bacterial killing, we performed opsonophagocytosis assays using murine macrophage J774A.1 cells. Both FpvA-KLH (33.9% killing) and WCV (72.7% killing) sera significantly increased opsonophagocytosis of *P. aeruginosa* PAO1 compared to sera from naïve animals (Figure 3E). These data show that antibodies generated by FpvA-KLH can mediate opsonophagocytosis *in vitro* and could potentially participate in bacterial clearance during infection.

### FpvA-KLH Vaccination Reduces the Bacterial Burden and Pulmonary Edema During Acute Pneumonia Caused by *P. aeruginosa*

**Figure 4:**
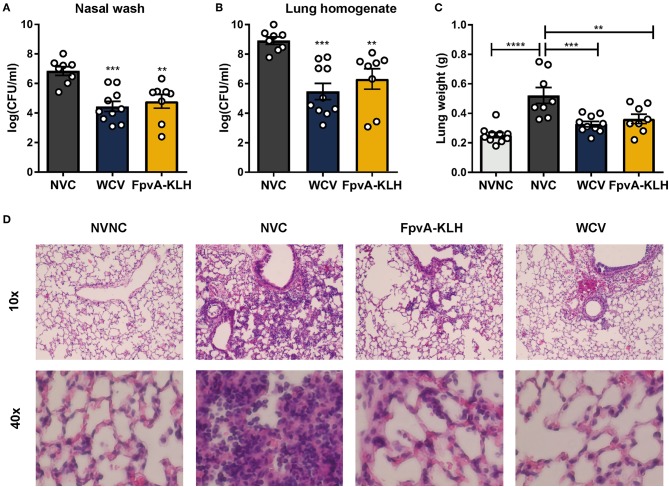
FpvA-KLH vaccination decreases bacterial burden and pulmonary edema in mice challenged with *P. aeruginosa*. The bacterial burden of NVC (*n* = 8), WCV (*n* = 10), and FpvA-KLH (*n* = 8) vaccinated mice in the nasal wash **(A)** and lung **(B)**. **(C)** Lung weight of vaccinated and challenged mice. The NVNC (*n* = 10) group was used as a control. Each circle represents data from one mouse. Data represent two independent experiments. The *p*-values were calculated using ANOVA followed by a Tukey's multiple-comparison test. Error bars are mean ± SEM values. The asterisks refer to statistical significance: ^**^p ≤ 0.01, ^***^p ≤ 0.001, ^****^p ≤ 0.0001. **(D)** H&E staining of lungs from immunized and adjuvant only control mice. Representative histopathological sections are shown (magnification = 10x and 40x). All data shown were collected at 16 h post-challenge.

To evaluate whether FpvA-KLH vaccination conferred protection against *P. aeruginosa*, mice were challenged on day 34 (Figure 2A). The bacterial burden in the nares and the lung were determined by plating the serial dilutions and counting viable bacteria at 16 h post-challenge. As expected, the bacterial load in the airways of the WCV mice was significantly lower than in NVC. Vaccination with FpvA-KLH resulted in decreased bacterial burden in nasal cavities and lung compared to the NVC control group (*p* = 0.0028 and *p* = 0.0061, respectively) (Figures 4A,B). This decrease was not significantly different between FpvA-KLH and WCV vaccinated mice, indicating that these mice are similarly protected against colonization in the lungs and nares. When the mice were vaccinated KLH alone, we did not observe any decrease in bacterial burden (data not shown). These results suggest that protection is conferred by the conjugation of the FpvA peptides to KLH and not KLH alone. Overall, vaccination with FpvA-KLH peptides leads to a significant decrease in the bacterial burden during acute *P. aeruginosa* pneumonia.

*P. aeruginosa* causes pneumonia associated with acute lung injury, inflammation, and pulmonary edema during infection (50, 51). We hypothesized that FpvA-KLH could help alleviate these pathologies. As an indicator of lung edema, we measured the wet weights of the lung from the infected mice. To observe pulmonary damage and inflammation, we performed lung histopathological analysis. The lung weight of mice vaccinated with FpvA-KLH was significantly decreased compared to the NVC mice after challenge. There were no significant differences in the lung weight between the FpvA-KLH vaccinated, NVNC, and WCV groups (Figure 4C). The body weight of mice was not significantly different in any of the groups (Figure S3), which suggests that the differences observed in lung weights are not associated with differences in overall body mass. In addition, the FpvA-KLH and WCV vaccinated mice had a lower recruitment of polymorphonucleated cells compared to NVC (Figure 4D). These data indicate that FpvA-KLH vaccination prevents the increase in pulmonary edema and recruitment of polymorphonucleated cells associated with *P. aeruginosa* infection. Altogether, these results show that FpvA-KLH vaccination reduces the bacterial burden in the lung and nares as well as lung damage and inflammation.

### Effect of FpvA-KLH Vaccination on the Cytokine and Myeloid Cell Response

**Figure 5:**
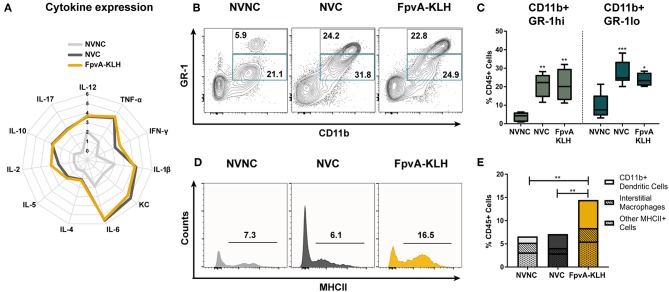
Innate immune responses in lungs at 16 h post-challenge. **(A)** Logarithmic average of overall cytokine responses in lung supernatant of NVNC, NVC, and FpvA-KLH vaccinated mice represented using a spider graph. The vertical axis shows the log scale increase of averaged cytokine response (pg/ml). **(B)** Representative contour plots of the NVNC (*n* = 5), NVC (*n* = 6), and FpvA-KLH (*n* = 4) groups showing the gating strategy for CD11b^+^GR-1^hi^ (neutrophils) and CD11b^+^GR-1^lo^ cell populations in lung leukocytes. **(C)** Proportion of CD11b^+^GR-1^hi^ and CD11b^+^GR-1^lo^ in lung leukocytes from **(B)**. Group comparisons were analyzed by ANOVA followed by a Tukey's multiple-comparison test. **(D)** Representative histograms show proportion of MHCII^+^ cells in CD45^+^SiglecF^−^GR-1^lo/−^CD11b^+^ cell populations. **(E)** MHCII^+^CD45^+^SiglecF^+/−^GR-1^lo/−^CD11b^+^ cell proportions of lung leukocytes. Subpopulations are separated as the proportion of CD11b^+^ dendritic cells (CD45^+^SiglecF^+/−^GR-1^lo/−^CD11b^+^CD64^−^CD24^+^) and interstitial macrophages (CD45^+^SiglecF^+/−^GR-1^lo/−^CD11b^+^CD64^+^CD24^−^) and other MHCII^+^ cells were shown. Group comparisons were analyzed by ANOVA followed by a Tukey's multiple-comparison test for MHCII^+^CD45^+^SiglecF^+/−^GR-1^lo/−^CD11b^+^ cell population. The asterisks refer to statistical significance: ^*^p ≤ 0.05, ^**^p ≤ 0.01, ^***^p ≤ 0.001.

Myeloid cells are crucial in the initial phase of infection. These cells participate in phagocytosis, antigen presentation, recruitment of other immune cells to the site of infection through cytokine secretion, and assist in inducing an adaptive immune response (52, 53). To understand how the FpvA-KLH vaccination mediates protection against *P. aeruginosa*, we characterized the immune response at 16 h post-challenge. We first measured the cytokines present in the lung. We observed that the amount of IL-17, IL-12, IL-5, IFN-γ, TNF-α, IL-1β, and IL-10 in the lung supernatant significantly increased in all challenged groups compared to non-challenged control mice (Figure S4, Table S3). However, there was no significant difference in cytokine secretion between the challenged groups, regardless of the vaccine they received (Figure 5A, Figure S4 and Table S3). From these experiments, we concluded that cytokine measurement during acute *P. aeruginosa* lung infection is insufficient to identify differences in the immune response of vaccinated and non-vaccinated mice in this model.

To gain further insights into the type of immune response mounted upon FpvA-KLH vaccination, we measured the number of circulating white blood cells and performed flow cytometry on lung single cell suspensions. We did not observe any differences in total white blood cells counts, or in the proportion of monocytes and neutrophils in blood (Figures S5A–C). However, we observed a significant increase in the percentage of eosinophils in the blood samples of the NVC group compared to NVNC and FpvA-KLH groups (Figure S5D). Interestingly, we detected opposite trends in the lung. First, we observed that the number of both CD11b^+^GR-1^hi^ (neutrophils) and CD11b^+^GR-1^lo^ myeloid cells increased in NVC and FpvA-KLH groups compared to the NVNC mice group (Figures 5B,C). However, we did not observe any significant changes between NVC and FpvA-KLH vaccinated groups (Figure 5C). In addition, while the proportion of eosinophils was increased in blood of NVC group, it was not significantly different in the lung samples of NVC, NVC, and FpvA-KLH (Figures S5D, S6A). Also, there were no significant differences in the proportion of lung alveolar macrophages between any of the challenged groups (Figure S6B).

### FpvA-KLH Vaccine Stimulates the Recruitment of Antigen-Presenting Cells to the Site of Infection

CD11b^+^ cells are of particular interest during vaccination and challenge as they include professional antigen presenting cells (APCs), which are crucial in the establishment of an adaptive immune response. To further determine the effect of the FpvA-KLH vaccination on MHCII^+^ CD11b^+^ cell population in the lungs, we used the gating strategy shown in Figure S1. We observed that MHCII^+^ cells in the CD45^+^SiglecF^+/−^GR1^lo/−^CD11b^+^ myeloid cell population were increased more than 2-fold in the FpvA-KLH vaccinated and challenged mice compared to NVC and NVNC (Figures 5D,E). Within this population, CD11b^+^ dendritic cells (CD45^+^SiglecF^+/−^GR-1^lo/−^CD11b^+^CD64^−^CD24^+^) were the major cell type increased in the FpvA-KLH vaccinated group compared to NVNC and NVC (Figure 5E and Figure S6C). There were no significant differences in the proportion of interstitial macrophages (CD45^+^SiglecF^+/−^GR-1^lo/−^CD11b^+^CD64^+^CD24^−^) between any of the challenged groups (Figure S6D). Overall, these results suggest that FpvA-KLH vaccination increases the recruitment of CD45^+^SiglecF^+/−^GR-1^lo/−^CD11b^+^MHCII^+^ myeloid cells to the lung upon challenge, and the majority of these cells are CD11b^+^ dendritic cells.

### Intranasal FpvA-KLH Vaccine With Curdlan Induces a Th17 Immune Response

**Figure 6:**
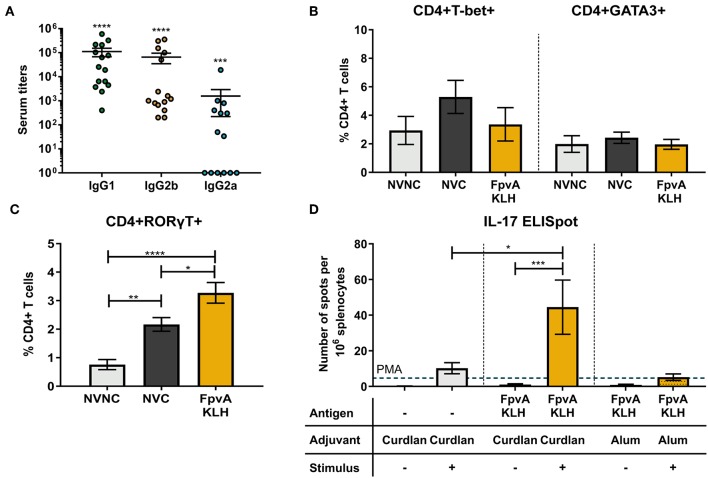
Quantification of the T cell immune response. **(A)** Elicited IgG subtypes (IgG1, IgG2b, and IgG2a) of FpvA-KLH vaccinated sera using the unconjugated FpvA peptides as antigen. Each circle represents data from one mouse. Data represent three independent experiments combined. *P*-values were calculated by comparing data to hypothetical value of 1, using a Wilcoxon signed-ranked test. Error bars are mean ± SEM values. In **(B,C)**, splenocytes were isolated from NVNC (*n* = 5), NVC (*n* = 8), and FpvA-KLH (*n* = 5) vaccinated groups 7 days post-challenge. **(B)** CD4^+^T-bet^+^ (Th1) and CD4^+^GATA3^+^ (Th2) cell proportions in the spleen. **(C)** Proportion of CD4^+^RORγT^+^ (Th17) cells in the spleen. **(D)** ELISpot assay with splenocytes to identify IL-17 specific response of each group in the presence or absence of heat-killed *P. aeruginosa* at day 34 (*n* = 5 in each group). Phorbol 12-myristate 13-acetate (PMA) was used as a positive stimulant control. Group comparisons were analyzed by ANOVA followed by a Tukey's multiple-comparison test. The asterisks refer to statistical significance: ^*^p ≤ 0.05, ^**^p ≤ 0.01, ^***^p ≤ 0.001, ^****^p ≤ 0.0001. Error bars are mean ± SEM values.

Th1 and Th17 specific immune responses were previously shown to decrease the severity of pneumonia caused by *P. aeruginosa* and are required to mount an adaptive immune response against this pathogen (33, 34). To characterize the type of CD4^+^ T cell response elicited by FpvA-KLH vaccination, we first identified the presence of IgG2 and IgG1 isotypes as surrogate markers of Th1 and Th2 immune responses, respectively. We observed the presence of FpvA peptide-specific IgG1 and IgG2 antibodies in the serum (Figure 6A). FpvA peptide-specific IgG2b antibodies were detected in all sera of FpvA-KLH vaccinated mice, while we detected IgG2a only in some of them (8/14) (Figure 6A). Interestingly, we did not observe any increase in the percentage of Th1 (CD4^+^Tbet^+^) or Th2 cells (CD4^+^GATA3^+^) in the splenocytes of FpvA-KLH vaccinated and challenge groups compared to NVNC or NVC at day 7 post-challenge (Figure 6B). However, we observed a significant increase in the Th17 (CD4^+^RORγT^+^) cell population in the FpvA-KLH vaccinated mice compared to both NVC and NVNC mice groups (Figure 6C). These data suggest that FpvA-KLH vaccination with curdlan leads to a Th17 cellular immune response.

To evaluate whether the elicited cellular immune response is antigen-specific, we performed IFN-γ and IL-17 ELISpot assays using splenocytes. In the mice vaccinated with curdlan alone or FpvA-KLH with curdlan, we did not observe any difference in IFN-γ secreting cells in response to stimulation with *P. aeruginosa* (Figure S7). On the other hand, splenocytes from FpvA-KLH vaccinated mice had a significantly higher number of IL-17 secreting cells than those immunized with curdlan alone in response to stimulation with *P. aeruginosa* (Figure 6D). Previous studies have shown that the adjuvant curdlan is an inducer of Th17 immune responses (35, 54). To determine whether the increase in the Th17 response is due to the inclusion of the FpvA-KLH peptides or the adjuvant, we re-formulated the FpvA-KLH vaccine with alum adjuvant. We observed that the FpvA-KLH vaccine adjuvanted with alum was not able to induce a significant increase in IL-17 producing cells in response to heat-killed *P. aeruginosa* compared to unstimulated control (Figure 6D). These results may indicate that both the adjuvant curdlan combined with the FpvA-KLH antigen are necessary to induce IL-17 secretion in splenocytes.

### Induction of Mucosal Memory Immune Response by Intranasal FpvA-KLH Vaccination

**Figure 7:**
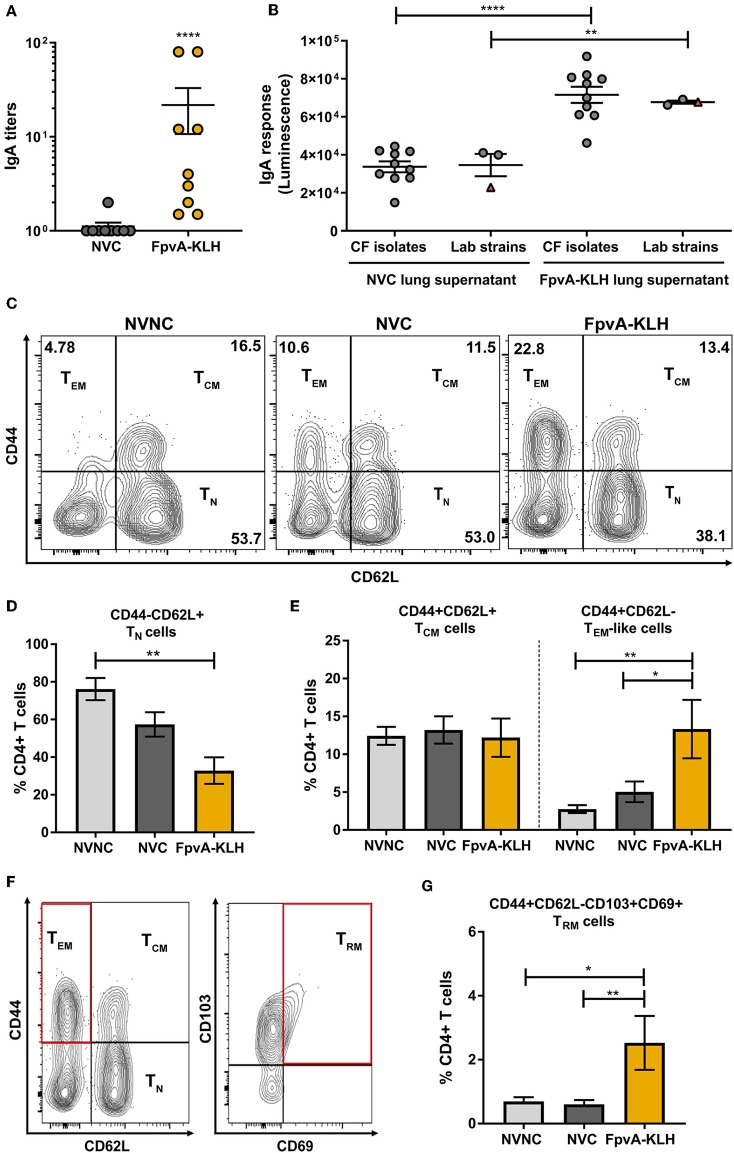
FpvA-KLH vaccine mucosal cellular and humoral immune responses in lung. **(A)** Lung supernatant IgA antibody titers of NVC (*n* = 9), and FpvA-KLH (*n* = 9) vaccinated and challenged mice using unconjugated FpvA peptides as antigen. **(B)** Detection of IgA binding from lung supernatants to *P. aeruginosa* lab-adapted (*n* = 3) and CF (*n* = 10) isolates. Each circle represents different *P. aeruginosa* strains, triangles show the PAO1 strain used for infection. **(C)** Representative contour plots of the NVNC (*n* = 5), NVC (*n* = 6), and FpvA-KLH (*n* = 4) groups showing the gating strategy for central memory (T_CM_), T effector memory-like (T_EM_), and naïve T cells (T_N_). **(D)** Proportion of naïve T cell population. **(E)** Proportion of central memory T cells and effector memory-like T cells. **(F)** Representative gating strategy for tissue-resident memory T cells (T_RM_). **(G)** Proportion of tissue-resident memory cells. Comparisons in **(A,B)** were performed using an unpaired student *t*-test. Group comparisons in **(D,E,G)** were analyzed by ANOVA followed by a Tukey's multiple-comparison test. The asterisks refer to statistical significance: ^*^p ≤ 0.05, ^**^p ≤ 0.01, ^****^p ≤ 0.0001. Error bars are mean ± SEM values.

Previous studies have shown that upon intranasal vaccine administration, IgA (38) and tissue-resident memory response can be elicited (55). These responses play an essential role in protecting against respiratory bacterial pathogens. Therefore, we hypothesized that intranasal vaccination with FpvA-KLH could induce a humoral mucosal immune response via IgA secretion as well as a tissue-resident memory response in upper airways. Mice vaccinated intranasally with FpvA-KLH had significant IgA titers against FpvA peptides compared to NVC mice in lung supernatants (Figure 7A). In addition, these IgA antibodies were able to bind significantly to different lab-adapted and CF *P. aeruginosa* isolates (Figure 7B). To determine whether FpvA-KLH vaccination also increased the recruitment of memory T cells to the lung, we performed flow cytometry. The contour plots show the gating strategy in NVNC, NVC, and FpvA-KLH groups to identify naïve CD4^+^ T cells (T_N_) (CD4^+^CD44^−^CD62L^+^), T effector memory-like cells (T_EM_) (CD4^+^CD44^+^CD62L^−^) and T central memory cells (T_CM_) (CD4^+^CD44^+^CD62L^+^) within CD4+ T cell population (Figure 7C). We observed that the proportion of naïve CD4^+^ T cells was decreased in FpvA-KLH vaccinated mice compared to NVNC (Figure 7D). While there are fewer naïve CD4^+^ T cells, we observed that the proportion of T effector memory-like cells (T_EM_) but not T central memory cells (T_CM_) (CD4^+^CD44^+^CD62L^+^) was significantly increased in the FpvA-KLH group compared to NVC and NVNC (Figure 7E). Among the T-effector memory-like cell population, the percentage of CD103^+^CD69^+^ cells (Figure 7F), which are known to have a tissue-resident memory phenotype (T_RM_) (55), was significantly increased in the FpvA-KLH vaccinated mice compared to NVNC and NVC (Figure 7G). Overall, the intranasal FpvA-KLH vaccine can induce mucosal IgA, as well as a tissue-resident memory T cell response in the lung. These results suggest that intranasal vaccination with FpvA-KLH elicits both humoral and cellular mucosal immune responses.

## Discussion

The ferripyoverdine receptor FpvA, is one of the main iron acquisition receptors of *P. aeruginosa* (15). It is essential for virulence and homeostasis, is expressed during infection, and is known to be antigenic (10, 12, 17, 19–21). In this study, we explored if a vaccine containing peptides based on the outer-membrane regions of FpvA could be protective against acute *P. aeruginosa* pneumonia in mice. We demonstrated that the FpvA peptides conjugated to the carrier protein KLH can induce both cellular and humoral immune responses and can provide protection against *P. aeruginosa* acute pneumonia in mice.

Peptide-based vaccines have been shown to be protective against both bacterial and viral pathogens in animal models (56). They can provide solutions with minimal cross-reactivity or reactogenicity in patients, as the type of response they trigger can be more finely controlled (57). Due to their small size, peptides are usually weakly immunogenic without carrier molecules to induce a robust immune response (56, 58). As expected, we observed that the FpvA peptides used in this study were more immunogenic when conjugated to the carrier protein KLH. Overall, antibodies secreted in response to the FpvA peptides conjugated to KLH were able to recognize the unconjugated FpvA peptides, the full-length FpvA protein, as well as whole bacteria *in vitro*. These data suggest that while we used short and linear antigen sequences for vaccination, the FpvA-KLH peptides were sufficient to provide a response that would also recognize the full protein and the bacteria.

Previous studies have identified the FpvA protein as a potential protective vaccine antigen for *P. aeruginosa* (16, 17). In an earlier study, purified FpvA protein adjuvanted with curdlan administered intranasally could produce antigen-specific antibody responses but failed to protect against *P. aeruginosa* (17). In this study, while we expressed and purified the FpvA protein in *E. coli*, we chose not to pursue its use as an antigen due to difficulties in purification and low solubility. Smaller peptides based on the outer-membrane regions of OMPs are more soluble and can be used as a substitute for the whole protein. In addition, biological impurities from purified OMPs, such as endotoxin, are another challenge to formulate clinically safe vaccines (58). Instead, we used extracellular peptides based on FpvA conjugated to KLH. We observed that vaccination with FpvA-KLH was as protective as WCV. Differences between the protection provided by FpvA-KLH peptides and the whole FpvA protein could be associated with protein conformation, folding, stability, and solubility. Interestingly, among the four selected FpvA peptides, FpvA peptide 2 was the most immunogenic peptide. The efficacy of using FpvA peptide 2 conjugated to KLH alone or other carrier proteins remains to be further examined.

Vaccine-mediated protection is often conferred by the production of antigen-specific antibodies that neutralize bacteria and their toxins to facilitate clearance (59). Vaccination with FpvA-KLH led to the production of IgM, IgG, and IgA in serum, and mucosal IgA in the lung. Both IgG from serum and IgA from lung supernatants were able to recognize lab-adapted strains as well as CF clinical *P. aeruginosa* isolates. We also showed that the antibodies present in serum were able to mediate opsonophagocytosis and participate in bacterial clearance *in vitro* suggesting a potential role for these antibodies during infection. Similar experiments could not be performed with lung IgA due to the low concentration of these antibodies in the samples. Interestingly, we did not find a correlation between antibody titers (IgM, IgG, or IgA) and bacterial CFU counts in vaccinated and challenged animals (data not shown). This may indicate that immunoglobulins alone are not sufficient to promote the clearance of *P. aeruginosa*, and that other components of the immune system have a role in protection. Additional studies are required to determine the exact role of antibodies, and in particular IgA, in the mechanism of protection of this mucosal vaccine.

In this study, we observed a strong cytokine response in mice challenged with *P. aeruginosa*, which is characteristic of infections caused by this bacterium (60, 61). Bacterial products drive inflammation in the lungs by interacting with pattern recognition receptors (PRR's) such as Toll-like receptors or NOD-like receptors (62, 63). In addition, the generation of additional bacterial products resulting from bacterial clearance also promote a pro-inflammatory response (61). Therefore, even though we observed decrease in bacterial burden in the FpvA-KLH vaccinated mice, we still detected a strong acute inflammatory cytokine response in the lungs. Future studies monitoring the cytokine response and cellular recruitment of cells over-time from bacterial challenge to clearance could help assess the differences in between the groups.

During our histopathology analysis, we observed a strong recruitment of immune cells to the lung of NVC mice, accompanied by an increase in lung weight. While this increase was in part due to the recruitment of neutrophils, no significant differences were observed in myeloperoxidase levels by ELISA or immunohistochemistry (data not shown). Interestingly, the only innate immune cell population that increased in the lung of FpvA-KLH vaccinated mice was MHCII^+^CD45^+^SiglecF^+/−^GR1^lo/−^CD11b^+^. Among this population, CD11b^+^ dendritic cells were the main cell type increased in FpvA-KLH vaccinated and challenged mice compared to NVC. One caveat of the flow cytometry analyses in this study is that mice were not perfused prior to organ harvest. As a result, there might be low remaining amounts of circulating blood in the tissue analyzed. To control for this, we measured the changes in white blood cells in the blood in response to challenge in vaccinated animals. We did not observe any significant changes in the proportion of neutrophils or monocytes in the blood in any of the vaccinated groups compared to control. For this reason, while we did not perform perfusions prior to lung single cell analysis, we do not anticipate that the presence of residual blood in the lung affected the proportion of cells measured in the lung. Murine lung CD11b^+^ dendritic cells are potent stimulators of Th17 immunity (64, 65). IL-17 production in acute pulmonary *P. aeruginosa* infections plays a protective role in the innate immune response to *P. aeruginosa* and is critical in vaccine-induced protection (16, 33). We observed that vaccination with FpvA-KLH leads to a significant increase in Th17 T cells and antigen-specific IL-17 secreting cells in the spleen. This result is not surprising as we used curdlan, which is known to promote a Th17 immune response (35, 54). Curdlan alone or alum-adjuvanted FpvA-KLH did not lead to production of an antigen-specific IL-17 response. This suggests that the combination of the FpvA-KLH peptides with the adjuvant curdlan is necessary to produce IL-17 secreting splenocytes. However, the exact mechanism of the IL-17 response elicited by FpvA-KLH vaccination and the effect of the adjuvants used requires further examination.

Intranasal vaccination induces protective systemic and mucosal humoral and cellular responses in the respiratory tract (38). After intranasal FpvA-KLH vaccination, we detected a significant increase in IgA response in the lung supernatant. This suggests that intranasal FpvA-KLH leads to humoral mucosal immunity. Previous studies indicate the importance of a CD4^+^ tissue-resident memory response for naturally-acquired immunity against bacterial infections (55). To assess whether tissue-resident memory cells were present in the lung of vaccinated animals, we performed flow cytometry to determine the proportion of T_N_, T_CM_, T_EM_, and T_RM_ cells. We observed a significant decrease in the proportion of T_N_ cells while the proportion of T_EM_ increased in FpvA-KLH vaccinated animals compared to NVNC and NVC controls. These changes might not be tissue specific and could be in part attributed to residual blood in the tissue. In addition, we observed that vaccination with FpvA-KLH leads to a significant increase in CD4^+^ tissue-resident memory T cells. Since the markers used in this study to identify tissue-resident memory T cells are not expressed in circulating T cells but only in tissue-resident memory T cells, we do not anticipate that the presence of residual blood in the tissue influenced the data presented here. Overall, this study shows the recruitment of tissue-resident memory T cells to the lung in response to intranasal vaccination; however, the role of these cells in protection against *P. aeruginosa* has not yet been demonstrated.

In this study, we examined the efficacy of FpvA-KLH vaccination in a murine model of acute pneumonia. The efficacy of the FpvA-KLH vaccine in the context of CF remains to be further examined. Unfortunately, the currently available CFTR mutant CF mice models do not recapitulate CF lung disease (66). However, the β-ENaC mouse model, which overexpress the β-subunit of the epithelial sodium channel (ENaC), displays CF-like clinical lung phenotype (67). In future studies, FpvA-KLH vaccination will be studied using the β-ENaC mouse model to evaluate vaccine efficacy on *P. aeruginosa*-induced lung disease.

Overall, we developed an intranasal peptide-based subunit *P. aeruginosa* vaccine using the main iron acquisition receptor of *P. aeruginosa*, FpvA, as a vaccine antigen. We observed that FpvA-KLH vaccination decreased the bacterial burden as well as lung edema. The FpvA-KLH vaccine was able to induce antigen-specific humoral and IL-17 immune response as well as a mucosal adaptive immune response in acute pneumonia. Taken as a whole, this study demonstrates that the peptide-based FpvA conjugate vaccine is an effective strategy in mice that could be used to prevent lung infections caused by *P. aeruginosa* in at-risk patients.

## Data Availability Statement

All datasets generated for this study are included in the manuscript/Supplementary Files.

## Ethics Statement

The animal study was reviewed and approved by West Virginia University Institutional Animal Care and Use Committees (WVU-ACUC protocol 1606003173).

## Author Contributions

ES-K and MB participated in peptide design, vaccine formulation, and performed histology analysis. ES-K, MB, and CB performed vaccine administration and bacterial challenge. MB, FD, and ES-K performed euthanasia and tissue necropsy. WW and TW helped with tissue processing. CB, ES-K, JB, and SB collected the serum and performed the bacterial counting. ES-K and DB performed flow cytometry panel design, and analysis was performed with assistance from MV. ES-K, DB, AM, and JH participated in cytokine, hematology, and ELISpot assays. ES-K, CB, AM, and JB completed the ELISA experiments. ES-K performed opsonophagocytosis assays. WW performed FpvA cloning, purification, and analysis. ES-K, FD, and MB participated in experimental design, data analysis, and the composition of this manuscript.

### Conflict of Interest

MB and FD hold a provisional patent application on the antigens described in this publication. The remaining authors declare that the research was conducted in the absence of any commercial or financial relationships that could be construed as a potential conflict of interest.
