# Supplementary material for: Intranasal Peptide-Based FpvA-KLH Conjugate Vaccine Protects Mice From Pseudomonas aeruginosa Acute Murine Pneumonia
Source: Front Immunol. 2019 Oct 23;10:2497. doi: 10.3389/fimmu.2019.02497 (PMC6819369; doi:10.3389/fimmu.2019.02497)
Supplement: Supplementary file 1 [file Data_Sheet_1.docx]

Supplementary Material

**Figure S1.** Gating strategy for flow cytometry analysis of myeloid cells in the lung. Single live cells were first gated for CD45^+^ cells. Alveolar macrophages (CD45^+^SiglecF^+^CD11c^+^) were gated out from CD45^+^ cells, and remaining cells were gated for CD11b and GR1 markers. The neutrophil (CD45^+^CD11b^+^GR1^hi^) population was then gated out, and the remaining cells were gated for eosinophils (CD45^+^SiglecF^+^CD11c^-^). After exclusion of neutrophils, alveolar macrophages and eosinophils, the remaining population was gated for CD11b^+^MHCII^+^ cells. These cells were further gated for interstitial macrophages (CD45^+^ CD11b^+^SiglecF^+/-^GR-1^lo/-^MHCII^+^CD64^+^CD24^-^) and CD11b^+^ dendritic cells (CD45^+^Cd11b^+^ SiglecF^+/-^GR1^lo/-^MHCII^+^CD64^-^CD24^+^).

**Figure S2.** Serum antibodies from the FpvA-KLH vaccinated and challenged mice against individual FpvA peptides. IgG antibody titers against individual peptides from sera of mice vaccinated with FpvA-KLH (n=9). (Peptide 2, *p*=0.0039). The comparisons were made to the hypothetical value of 1 (no detectable response), using the Wilcoxon signed ranked test. The asterisks refer to the level of significance of each group compared to 1: ***p* ≤ 0.01.

**Figure S3:** Total body weight at 16 hours post-challenge. Total body weight of NVC (n=8), WCV (n=10), and FpvA-KLH (n=8) vaccinated and challenged mice. The NVNC (n=10) group was used as a control. Each circle represents data from one mouse. Data represent two independent experiments. Error bars are mean ± SEM values.


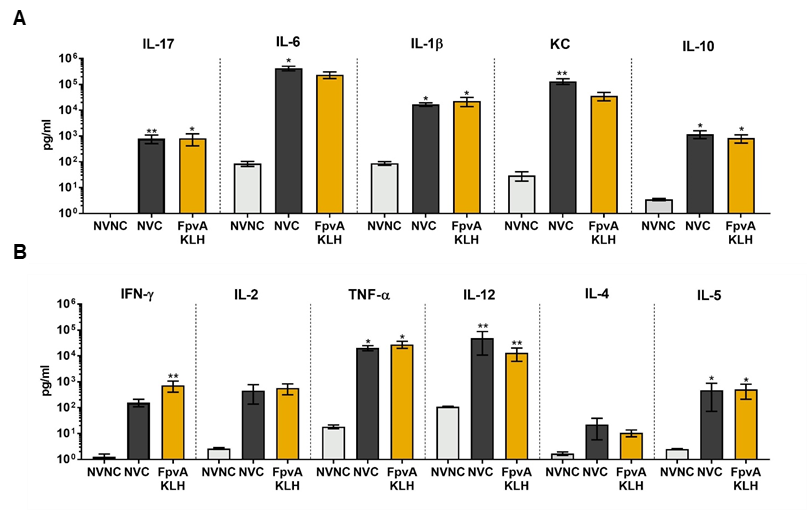


**Figure S4.** Analysis of cytokines in lung homogenates after challenge. Cytokines IL-17, IL-6, IL-1β, KC and IL-10 (**A)** IFN-γ, IL-2, TNF-α, IL-12, IL-4 and IL-5 (**B)** were quantified using electrochemiluminescence immunoassays. Group comparisons were performed using the Kruskal Wallis test. The asterisks refer to the level of significance: **p* ≤ 0.05; ***p* ≤ 0.01. Error bars are mean ± SEM values.

**Figure S5.** Total white blood cells and frequency of myeloid cell populations in blood 16 h post-challenge. Total white blood cells **(A)**, proportion of neutrophils **(B)**, monocytes **(C)**, and eosinophils **(D)** in NVNC (n=6), NVC (n=8), and FpvA-KLH (n=9) vaccinated mice. Group comparisons were analyzed by ANOVA followed by a Tukey’s multiple-comparison test. The asterisks refer to the level of significance: **p ≤*0.05; ***p ≤*0.01. Error bars are mean ± SEM values.

**
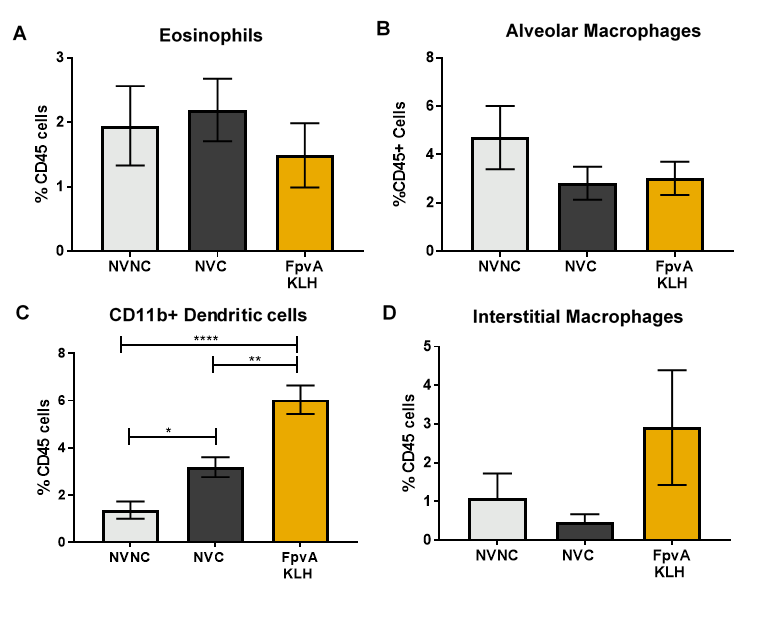
Figure S6.** Frequency of myeloid cell populations in lung 16 h post-challenge. The proportion of eosinophils **(A)**, alveolar macrophages **(B)**, CD11b^+^ dendritic cells **(C)**, and interstitial macrophages **(D)** in NVNC (n=5), NVC (n=6) and FpvA-KLH (n=4) vaccinated mice. Group comparisons were analyzed by ANOVA followed by a Tukey’s multiple-comparison test. The asterisks refer to the level of significance: **p ≤*0.05; ***p ≤*0.01; *****p ≤*0.0001. Error bars are mean ± SEM values.

**IFN-γ ELISPOT**

**
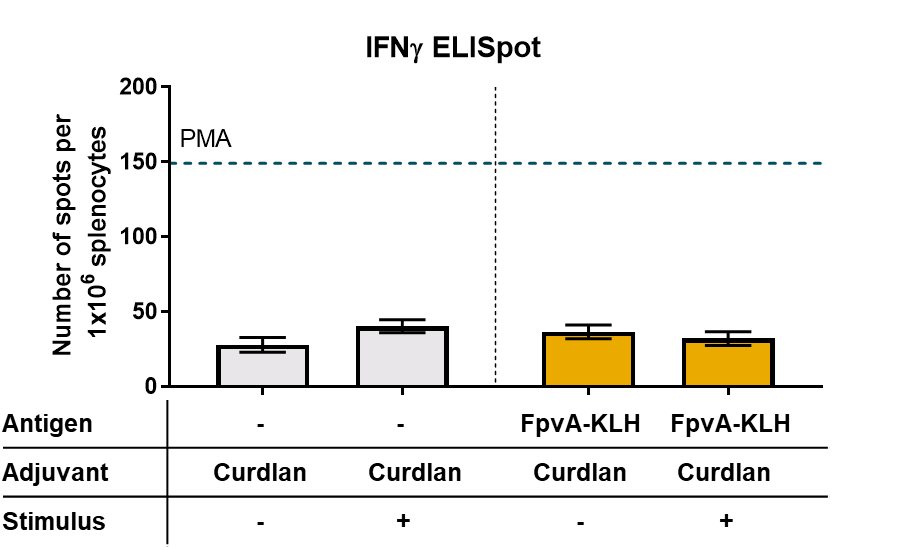
**

**Figure S7.** Antigen-specific IFN-γ response upon FpvA-KLH vaccination. ELISpot assay to identify IFN-γ specific response of intranasal curdlan only and FpvA-KLH vaccination with curdlan in the presence or absence of heat-killed *P. aeruginosa* at day 34 post-vaccination (n=5 in each group). Splenocytes were incubated with heat-killed whole-cell 2x10^7^ CFU/well *P. aeruginosa* or complete RPMI 1640 media without stimulant for 72 hours. PMA was used as a positive stimulant control. Group comparisons were analyzed by ANOVA followed by a Tukey’s multiple-comparison test.

**Table S1: PCR primers used to amplify *fpvA* gene.**

| PCR Primers | |
| --- | --- |
| Name | **Sequence 5’-3^’^** |
| *fpvA*-forward | AGTCGAGCTCATGGCAGCACCACACGGTCTCAG |
| *fpvA*-reverse | AGTCAAGCTTTTAATGATGATGATGATGATGGAAGTCCCAGCGAGTGCT |

**Table S2: Flow cytometry antibodies and panels used in this study.**

| Myeloid Panel | | | |
| --- | --- | --- | --- |
| Antibody | **Fluorophore** | **Company** | **Catalog Number** |
| CD45 | PE-CF594 | BD Biosciences | 562420 |
| CD11b | BV510 | BD Biosciences | 562950 |
| CD11c | APC-Cy7 | Biolegend | 117324 |
| CD24 | BV421 | BD Biosciences | 562563 |
| CD64 | APC | Biolegend | 139306 |
| GR-1 | PE | BD Biosciences | 553128 |
| MHCII | FITC | Thermo Fisher | MA1-10403 |
| Siglec-F | APC-R700 | BD Biosciences | 565183 |
| Intracellular T Cell Panel | | | |
| CD4 | APC-Cy7 | Biolegend | 100526 |
| CD8 | PerCP-Cy5.5 | BD Biosciences | 551162 |
| GATA3 | APC | Miltenyi Biotec | 130-100-650 |
| RoRγT | PE-CF594 | BD Biosciences | 562684 |
| T-bet | PE | BD Biosciences | 561265 |
| Resident Memory T Cell Panel | | | |
| CD4 | APC-Cy7 | Biolegend | 100526 |
| CD44 | BB515 | BD Biosciences | 564587 |
| CD62L | APC | BD Biosciences | 553152 |
| CD69 | BV421 | BD Biosciences | 562920 |
| CD103 | APC-R700 | BD Biosciences | 565529 |

**Table S3**: **The average of cytokines in lung supernatant at 16 hours post challenge.**

| Cytokine | NVNC | NVC | FpvA-KLH |
| --- | --- | --- | --- |
| IFN-γ | 1.3 ± 0.7 | 181.3 ± 160.7 | 639.6 ± 631.5 |
| IL-10 | 3.5 ± 0.7 | 1289.8 ± 850.9 | 1071.7 ± 1078.8 |
| IL-12 | 108.4 ± 8.8 | 4106.0 ± 1111.4 | 4932.1 ± 3452.8 |
| TNF-α | 18.3 ± 6.0 | 20931.1 ± 10359.3 | 28751.6 ± 21773.9 |
| IL-1β | 87.4 ± 27.8 | 18553.3 ± 6976 | 13220.0 ± 9423.3 |
| KC | 17.9 ± 3.6 | 132435.3 ± 80388.1 | 52429.2 ± 23586.7 |
| IL-6 | 67.3 ± 13.4 | 421386.9 ± 205115.9 | 332129.2 ± 96537 |
| IL-4 | 1.7 ± 0.5 | 13.5 ± 2.8 | 16.9 ± 6.4 |
| IL-5 | 2.5 ± 0.2 | 49.1 ± 19.6 | 76.7 ± 54.1 |
| IL-2 | 2.6 ± 0.4 | 118.6 ± 42.9 | 762.6 ± 735.1 |
| IL-17 | 0.9 ± 0.6 | 797.8 ±770.1 | 645.9 ± 1087.7 |

Data in the table represent the mean cytokine response ± standard error of the mean. Results are shown in pg/ml.
